# Multimodal assessment of right ventricle overload-metabolic and clinical consequences in pulmonary arterial hypertension

**DOI:** 10.1186/s12968-021-00743-2

**Published:** 2021-05-10

**Authors:** Remigiusz Kazimierczyk, Lukasz A. Malek, Piotr Szumowski, Stephan G. Nekolla, Piotr Blaszczak, Dorota Jurgilewicz, Marcin Hladunski, Bozena Sobkowicz, Janusz Mysliwiec, Ryszard Grzywna, Wlodzimierz J. Musial, Karol A. Kaminski

**Affiliations:** 1 Department of Cardiology, Medical University of Bialystok, Białystok, Poland; 2 Department of Epidemiology, Cardiovascular Disease Prevention and Health Promotion, National Institute of Cardiology, Warsaw, Poland; 3 Laboratory of Molecular Imaging, Medical University of Bialystok, Białystok, Poland; 4 Department of Nuclear Medicine, Medical University of Bialystok, Białystok, Poland; 5 Department of Nuclear Medicine, Technical University Munich, Munich, Germany; 6 Department of Cardiology, Cardinal Wyszynski’ Hospital, Lublin, Poland; 7 Department of Population Medicine and Lifestyle Diseases Prevention, Medical University of Bialystok, Waszyngtona 13a, Białystok, 15-269 Poland

**Keywords:** Primary pulmonary hypertension, Cardiovascular magnetic resonance, Positron emission tomography, Prognosis, Coupling

## Abstract

**Background:**

In pulmonary arterial hypertension (PAH) increased afterload leads to adaptive processes of the right ventricle (RV) that help to maintain arterio-ventricular coupling of RV and preserve cardiac output, but with time the adaptive mechanisms fail. In this study, we propose a multimodal approach which allows to estimate prognostic value of RV coupling parameters in PAH patients.

**Methods:**

Twenty-seven stable PAH patients (49.5 ± 15.5 years) and 12 controls underwent cardiovascular magnetic resonance (CMR). CMR feature tracking analysis was performed for RV global longitudinal strain assessment (RV GLS). RV-arterial coupling was evaluated by combination of RV GLS and three proposed surrogates of RV afterload—pulmonary artery systolic pressure (PASP), pulmonary vascular resistance (PVR) and pulmonary artery compliance (PAC). 18-FDG positron emission tomography (PET) analysis was used to assess RV glucose uptake presented as SUV_RV/LV_. Follow-up time of this study was 25 months and the clinical end-point was defined as death or clinical deterioration.

**Results:**

Coupling parameters (RV GLS/PASP, RV GLS/PVR and RV GLS*PAC) significantly correlated with RV function and standardized uptake value (SUV_RV/LV_). Patients who experienced a clinical end-point (n = 18) had a significantly worse coupling parameters at the baseline visit. RV GLS/PASP had the highest area under curve in predicting a clinical end-point and patients with a value higher than (−)0.29%/mmHg had significantly worse prognosis. It was also a statistically significant predictor of clinical end-point in multivariate analysis (adjusted R^2^ = 0.68; p < 0.001).

**Conclusions:**

Coupling parameters are linked with RV hemodynamics and glucose metabolism in PAH. Combining CMR and hemodynamic measurements offers more comprehensive assessment of RV function required for prognostication of PAH patients.

*Trial Registration*: NCT03688698, 09/26/2018, retrospectively registered; Protocol ID: 2017/25/N/NZ5/02689

## Introduction

Pulmonary arterial hypertension (PAH) is a progressive disease, in which declining right ventricular (RV) function with accompanying uncoupling to the pulmonary circulation is a turning point of clinical worsening. Therefore, accurate assessment of RV systolic function, especially before significant clinical deterioration is crucial for PAH patients [1–3]. The RV adapts to the increasing vascular load by enhancing contractility according to Frank-Starling law, preserving RV-arterial coupling and maintaining blood flow to peripheral demand [4, 5]. The concept of coupling mainly refers to the relationship between ventricular contractility and afterload thus alterations of this phenomenon may be an early marker of RV failure [6]. The gold standard to assess RV-arterial coupling requires invasive procedure to obtain pressure–volume loop-derived end-systolic elastance (Ees) to arterial elastances (Ea) ratio. This ratio (Ees/Ea) gives direct quantification of RV-arterial coupling [4]. However, measuring these parameters via pressure–volume loops is technically demanding and cannot be performed routinely during regular PAH patients’ assessments.

Cardiovascular magnetic resonance (CMR) imaging is the gold standard, noninvasive method for RV functional assessment. RV contractility may be described by various CMR parameters like RV ejection fraction (RVEF), tricuspid annular plane systolic excursion (TAPSE), fractional area change (FAC) or myocardial strain [7–9]. RV global longitudinal strain (GLS) obtained from CMR feature tracking was recently associated with worse outcomes in PAH [10–12]. RV GLS seems to be less load independent than RVEF. It also correlates with diastolic stiffness, and thus could reflect better RV contractility in overloaded RV in PAH [13, 14].

The RV afterload is composed of static and pulsatile components. According to Windkessel model, pulmonary arterial resistance (PVR) or pulmonary artery pressures represent only the mean resistive or static afterload (an opposition to forward flow) when the pulsatile component may be represented by pulmonary artery compliance parameter—PAC [15].

Combining RV contractility with estimations of its afterload, especially using multimodal techniques, seems to be a novel approach allowing not only to better understand RV hemodynamics but also creates possibilities of new prognostic markers.

In PAH, the RV also undergoes a number of metabolic changes, including the shift to the less energetically efficient process of glycolysis [3]. This results in a compensatory upregulation of glucose flux into RV myocytes. The flux can be quantified in positron emission tomography (PET) imaging and presented as ^18^F-fluorodeoxyglucose (FDG) standardized uptake value (SUV) parameter [16]. In severe PAH patients, due to higher pressure overload of RV maladaptive increase of the FDG uptake is observed [17]. Recently, we confirmed that FDG uptake (presented as SUV_RV/LV_) correlates with PAH progression and unfavourable outcomes [18].

In this research, we chose CMR-derived RV GLS as the main parameter of RV contractility. As surrogates of a RV afterload, we used systolic pulmonary artery pressure (PASP); PVR and PAC, all obtained from right heart catheterization (RHC) and CMR.

The aim of this study was to evaluate whether relating RV shortening, as assessed by feature tracking CMR, to the force developed, as expressed by PASP/PVR/PAC, could translate into a more accurate estimation of RV performance status and, consequently, in an improvement in risk prediction accuracy. We also hypothesized that presented above coupling parameters may be related to alterations in RV glucose uptake both preceding clinical deterioration.

## Methods

### Population characteristics

We enrolled 27 clinically stable, adult patients diagnosed with PAH in World Health Organization (WHO) class II or III. The diagnosis of pre-capillary pulmonary hypertension (PH) was confirmed by RHC [mean pulmonary artery pressure (mPAP) ≥ 25 mmHg, pulmonary artery wedge pressure (PAWP) ≤ 15 mmHg] and the use of an algorithm that included a perfusion lung scan, echocardiography, respiratory function tests, and computer tomography to exclude secondary PH causes according to European guidelines [1]. The exclusion criteria were following: patients in grade IV WHO class, Eisenmenger physiology, PAH associated with prevalent systemic-to-pulmonary shunts due to moderate to large defects (according to European guidelines) [1], group II, III, IV, V of PH and contraindications to CMR. The control group consisted of 12 healthy subjects who were selected based on sex and age. During the baseline evaluation, we performed a physical examination, six-minute walk test (6MWT), laboratory tests e.g. serum B-type natriuretic peptide (BNP), blood count and renal function parameters.

RHC was carried out only in the PAH group with a standard technique within median 4 [2–6] days of PET/CMR scans using a balloon-tipped 7F pulmonary artery catheter to assess systolic pulmonary artery pressure (sPAP), diastolic pulmonary artery pressure (dPAP), mPAP and PAWP. Cardiac output (CO) was measured by thermodilution method. PVR was calculated with the formula PVR = (mPAP-PAWP)/(CO) and expressed in Wood’s units (WU) and PAC parameter with formula PAC = stroke volume (SV)/(sPAP-dPAP). RV-arterial coupling was evaluated by the ratio of RV GLS/PASP; RV GLS/PVR; RV GLS*PAC.

Clinical follow-up was 25 months. Death, WHO class worsening, hospitalisation due to PH or right heart failure as assessed by European Society of Cardiology (ESC) criteria were used as composite clinical endpoint (CEP) for Kaplan–Meier analysis. All enrolled patients were re-hospitalized only in our Department and their clinical state or death were evaluated/confirmed by PAH specialists (co-authors of this paper). The study was approved by the local Bioethics Committee at Medical University of Bialystok (R-I-002/140/2017). All patients gave written informed consent for participating in the study, including taking and storage of blood samples. The investigation conforms with the principles outlined in the Declaration of Helsinki” (Br Med J 1964; ii: 177).

### PET/CMR imaging

Simultaneous PET/CMR imaging was performed with a 3T Biograph mMR hybrid system (Siemens, Healthineers Erlangen, Germany) at the baseline visit. CMR studies were analysed using a dedicated workstation and software (Horos v 3.3.5 with validated MRHeart plugin) [19]. CMR results were not known to treating physician and did not affect the decision about the therapy, simultaneously physician analyzing the CMR was not aware of the patient’s clinical state.

Systolic function assessment was based on cine balanced steady state free precession (bSSFP) short axis images from the tricuspid valve insertion point to the apex to encompass the entire RV. The imaging parameters were the following: field of view 360 mm, matrix 256 × 256, repetition time approximately 40.7 ms, echo time 1.49 ms, flip angle 50 degrees, slice thickness 6 mm, gap 1.2 mm, in-plane image resolution 1.4 × 1.4 × 6 mm^3^, temporal resolution 25 phases per cardiac cycle.

Short axis bSSFP cine images were initially previewed from the base to the apex in a cinematic mode; then endocardial and epicardial contours for RV end-diastole and end-systole were manually traced. Trabeculae were considered as ventricle cavities. The delineated contours were used for the quantification of RV variables, which were then indexed to body surface area (BSA). RV FAC was obtained from formula (end-diastolic area – end-systolic area)/end-diastolic area × 100.

RV strain analysis was performed with the use of a dedicated software (QStrain, Mass Medis, Leiden, The Netherlands). For the purpose of analysis endocardial borders of the RV were manually traced in end-systole and end-diastole on 4-chamber view and used for generation of RV GLS [14].

Heart glucose metabolism was assessed with FDG as a tracer in PET. One hour after *iv* injection of FDG (185 MBq ± 15 MBq), myocardial PET imaging was performed as previously described [18]. Its uptake was quantified as mean SUV for both the left ventricle (LV) and RV and presented as a ratio of SUV_RV/LV_.

### Statistical analysis

The distribution of the variables was checked using the Kolmogorov–Smirnov test. The data are expressed as a mean ± standard deviation (SD) or median [interquartile range] as appropriate. Statistical analysis was performed using Student’s *t*-test or Mann–Whitney U test for continuous data depending on distribution. Spearman’s correlation coefficient was used to examine the relationship between two continuous variables. Benjamini–Hochberg correction was used to account for multiple comparisons in correlation analysis. Univariable and multivariable Cox proportional hazards regression analyses were performed to identify independent variables associated with end-point. Receiver operator characteristic curves (ROC) were plotted to determine the area under the curve (AUC) and sensitivity and specificity of the optimal cut-offs (binomial method). DeLong’s test was used to compare two AUC results. To investigate the occurrence of clinical endpoints Kaplan Meier method with log-rank test was implemented. p < 0.05 was deemed statistically significant. A statistical software package STATA13 (Stata Corporation, College Station, Texas, USA) was used for the analysis.

## Results

### Patients’ characteristics

Twenty-seven stable PAH patients 49.5 ± 15.5 years (idiopathic/heritable n = 18; connective tissue diseases n = 4; associated with congenital small/coincidental defects n = 5) and 12 healthy subjects (control group) selected based on age and sex (44.8 ± 13.5 years, 8 females) were enrolled in the study. Five PAH patients (18%) were incident cases while the rest of the study group were prevalent—receiving PAH specific treatment at the time of survey. Most of them were in the WHO functional Class III (62%, n = 17) and 10 patients (38%) were in WHO functional Class II; majority of patients were women (62%, n = 17). According to 1-year mortality risk groups presented in ESC guidelines [7], nineteen patients (70%) were at intermediate risk; five patients (19%) at low risk and three patients (11%) at high risk. The mean 6-min walk test distance was 387 ± 103 m and median plasma BNP level was 269 pg/ml. Groups characteristics’ and PET/CMR and RHC results are presented in Table 1.

**Table 1:** Basic characteristics of pulmonary arterial hypertension (PAH) group and healthy controls

	Pulmonary artery hypertension	Healthy controls
Subjects, n	27	12
Age, years	49.5 ± 15.5	44.7 ± 13.5
Sex (females), % (n)	62 (17)	67 (8)
BSA, m^2^	1.8 ± 0.2	1.8 ± 0.2
6MWT, m	387 ± 103	
BNP, pg/ml	269 [22–925]	
*Aetiology*		
IPAH/HPAH, % (n)	66 (18)	
CTDPH, % (n)	14 (4)	
CHDPH, % (n)	20 (5)	
*Therapy*		
PDE5 inhibitors, % (n)	40 (11)	
ERA, % (n)	11 (3)	
Prostacyclins, % (n)	20 (5)	
Dual PDE5 inhibitor + ERA, % (n)	29 (8)	
*Hemodynamics*		
sPAP, mmHg	77.4 ± 27.7	
dPAP, mmHg	32.3 ± 14.1	
mPAP, mmHg	48.6 ± 17.6	
PCWP, mmHg	10.3 ± 2.4	
DPG, mmHg	22.9 ± 13.5	
PVR, Wood Units	8.7 ± 5.4	
CI, L/min/m^2^	2.7 ± 0.8	
RAP, mmHg	8.6 ± 3.5	
*RV functional parameters (CMR)*		
RVEF, %	44.8 ± 10.2^	63.8 ± 5.8
TAPSE, mm	19 ± 4.3^	24.9 ± 2.4
RV EDV/BSA, ml/m^2^	117.8 ± 29.9^	73.6 ± 12.2
RV ESV/BSA, ml/m^2^	66.5 ± 27.1^	28.2 ± 9.6
RV mass/BSA, g/m^2^	42.9 ± 17.1^	23.8 ± 4.9
PAC, ml/mmHg	2.5 ± 1.6	
GLS, %	− 16.4 ± 7.4^	− 30.7 ± 9.7
*Myocardial metabolism (PET)*		
SUV_RV_/SUV_LV_ ratio	1.03 ± 0.76^	0.19 ± 0.08

Data are presented as mean ± standard deviation or median [interquartile range]

*6MWD* 6-minute walk test distance, *BSA* body surface area, *BNP* brain natriuretic peptide, *CI* cardiac index, *CHDPAH* congenital heart disease related pulmonary arterial hypertension, *CMR* cardiovascular magnetic resonance, *CTDPAH* connective tissue disease related pulmonary arterial hypertension, *DPG* diastolic pulmonary gradient, *dPAP* diastolic pulmonary artery pressure, *EDV* end-diastolic volume, *ESV* end-systolic volume, *ERA* endothelin receptor antagonist, *GLS* global longitudinal strain, *HPAH* heritable pulmonary arterial hypertension, *IPAH* idiopathic pulmonary arterial hypertension, *GLS* global longitudinal strain, *mPAP* mean pulmonary artery pressure, *PAC* pulmonary arterial compliance, *PAH* pulmonary arterial hypertension, *PASP* pulmonary artery systolic pressure, *PCWP* pulmonary capillary wedge pressure, *PDE5* phosphodiesterase type 5, *PET* positron emission tomography, *PVR* pulmonary vascular resistance, *sPAP* systolic pulmonary artery pressure, *RAP* right atrial pressure, *RV* right ventricle, *RVEF* right ventricle ejection fraction, *SUV* standardized uptake value, *SvO*_*2*_ mixed venous oxygen saturation, *TAPSE* cardiac magnetic resonance tricuspid annular plane systolic excursion, *WHO* World Health Organisation

^Statistically significant difference between PAH and control groups, p < 0.005

### CMR parameters and RV function

**Fig. 1:**
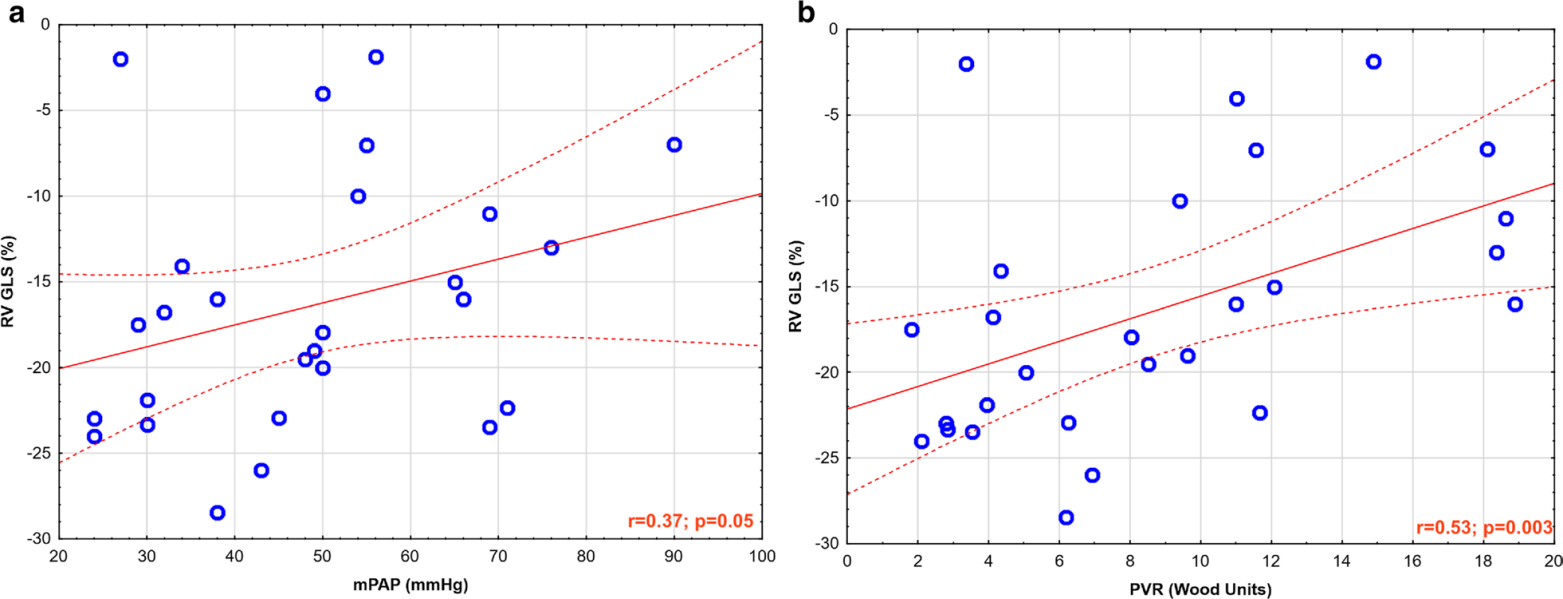
Spearman correlations between right ventricular (RV) global longitudinal strain (GLS) (from cardiovascular magnetic resonance (CMR)) and **a** mean pulmonary artery pressure (mPAP) and **b** pulmonary vascular resistance (PVR), both obtained from right heart catheterization (RHC) in the pulmonary artery hypertension (PAH) group. *CMR* cardiac magnetic resonance; *GLS* global longitudinal strain; *mPAP* mean pulmonary arterial hypertension; *PAH* pulmonary arterial hypertension; *PVR* pulmonary vascular resistance, *RHC* right heart catheterization; *RV* right ventricle.

In the PAH group, RV dimensions and mass were significantly higher than in the healthy control group and RVEF was significantly lower (Table 1). LV ejection fraction (LVEF) did not differ between both groups. RV function presented as RV GLS was significantly less negative (worse) in PAH group than in the healthy control group (− 16.4 ± 7.4% vs − 30.7 ± 9.7%, p < 0.001). In the study group, RV GLS correlated with hemodynamic parameters from RHC – mPAP (r = 0.37, p = 0.05) and PVR (r = 0.53, p = 0.003), Fig. 1; from CMR – with TAPSE (r = − 0.54, p = 0.01) and RVEF (r = − 0.7, p < 0.001).

Mean values of the RV-pulmonary artery coupling parameters in PAH group were—RV GLS/PASP – (−)0.25 ± 0.17%/mmHg; RV GLS/PVR – (−)3.3 ± 2.8%/WU and RV GLS*PAC – (−)42.9 ± 32.1%*mmHg/ml.

All three parameters significantly correlated with established CMR-derived RV systolic function variables—RVEF and FAC (Table 2). Interestingly, three coupling parameters correlated also with BNP levels but not with functional PAH prognosis predictors—WHO class and 6MWT.

**Table 2:** Spearman correlations between RV-arterial coupling parameters and other parameters of RV function and metabolism

	RV GLS/PASP	RV GLS/PVR	RV GLS*PAC
WHO class	r = 0.28; p = 0.15	r = 0.28; p = 0.14	r = 0.26; p = 0.19
6MWT distance	r = − 0.22; p = 0.25	r = − 0.29; p = 0.13	r = − 0.22; p = 0.24
BNP level	r = 0.31; p = 0.10	r = 0.34; p = 0.07	r = 0.30; p = 0.12
TAPSE (CMR)	r = − 0.15; p = 0.43	r = − 0.15; p = 0.42	r = − 0.08; p = 0.65
RVEF (CMR)	r = − 0.75; p = 0.000005^	r = − 0.71; p = 0.00002^	r = − 0.73; p = 0.00001^
FAC (CMR)	r = − 0.78; p = 0.000002^	r = − 0.77; p = 0.000001^	r = − 0.69; p = 0.00004^
RV mass/BSA (CMR)	r = 0.59; p = 0.001^	r = 0.60; p = 0.0008^	r = 0.47; p = 0.012^
CI (RHC)	r = − 0.54; p = 0.003^	r = − 0.63; p = 0.0004^	r = − 0.48; p = 0.01^
SUV_RV/LV_ (PET)	r = 0.67; p = 0.0002^	r = 0.55; p = 0.002^	r = 0.69; p = 0.00006^

*6MWT* six minute walk test distance, *BNP* serum brain natriuretic peptide, *BSA* body surface area, *CI* cardiac index, *CMR* cardiac magnetic resonance, *GLS* global longitudinal strain, *FAC* fractional area change, *LV* left ventricle, *PAC* pulmonary arterial compliance, *PASP* pulmonary artery systolic pressure, *PET* positron emission tomography, *PVR* pulmonary vascular resistance, *RV* right ventricle, *RVEF* right ventricle ejection fraction, *SUV* standardized uptake value, *TAPSE* tricuspid annular plane systolic excursion, *WHO* World Health Organisation

^p-value significant (lower than 0.05) after Benjamini–Hochberg correction

### RV—coupling and RV metabolism

In PET analysis, the cardiac FDG uptake parameter SUV_RV/LV_ was significantly higher in PAH group than in healthy controls (1.03 ± 0.76 vs 0.19 ± 0.08, p < 0.001). It also correlated with parameters of RV dysfunction e.g. RVEF (r = − 0.52, p < 0.001) and mPAP (r = 0.77, p < 0.001).

**Fig. 2:**
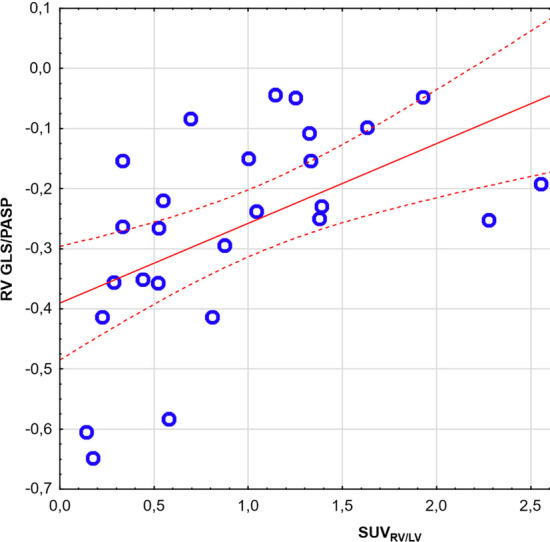
Spearman correlation between RV GLS/PASP and SUV_RV/LV_ PAH group. *GLS* global longitudinal strain; *LV* left ventricle; *PAH* pulmonary arterial hypertension; *PASP* pulmonary artery systolic pressure; *RV* right ventricle; *SUV* standardized uptake value.

All three coupling parameters (RV GLS/PASP, RV GLS/PVR and RV GLS*PAC) significantly correlated with SUV_RV/LV_, Table 2, Fig. 2. Importantly, no significant correlation between non-indexed RV GLS and SUV_RV/LV_ was found (r = 0.36, p = 0.11).

In univariate logistic regression analysis, RV GLS, RV GLS/PASP, WHO class, 6MWT distance, CVP were associated with SUV_RV/LV_ dichotomized at 1 (higher glucose uptake of RV than of LV), which value has also significant prognostic value [16, 18].

### Survival analysis

After 25 months, 18 patients (66%) met a composite endpoint. Five patients died, 13 patients had WHO class worsening (ten patients from WHO class II to III and three patients from WHO class III to IV) including four patients who required initiation of parenteral prostacyclin analogue. Mean time to clinical worsening was 16.9 ± 8.0 months.

Patients who reached CEP had a significantly higher RV GLS and all three coupling parameters at the baseline visit than patients who remained stable (Table 3). There were also significant differences in CMR and RHC-derived parameters but not in 6MWT distance or BNP levels. In comparison to coupling parameters difference in non-indexed RV afterload surrogate—PAC was not statistically significant.

**Table 3:** Comparison between patients with composite end-point (CEP+) and without (CEP−)

	CEP (+) patients	CEP (−) patients	p-value
Patients, n	18	9	
BNP, pg/ml	241 [22–925]	62 [36–435]	0.19
6MWT distance, m	358.8 ± 97.2	443.6 ± 95.4	0.06
SUV_RV_/SUV_LV_ ratio	1.29 ± 0.78	0.51 ± 0.39	0.003
RVEF, %	40.9 ± 8.8	52.5 ± 8.4	0.004
TAPSE, mm	17.9 ± 4.0	21.3 ± 4.2	0.07
PAC, ml/mmHg	2.0 ± 0.9	3.3 ± 2.3	0.06
mPAP, mmHg	56.3 ± 15.8	33.1 ± 8.2	< 0.001
PVR, wood units	10.9 ± 5.3	4.3 ± 2.3	< 0.001
RV GLS, %	− 13.5 ± 7	− 22.2 ± 3.8	0.001
RV GLS/PASP, %/mmHg	− 0.16 ± 0.08	− 0.44 ± 0.11	< 0.001
RV GLS/PVR, %/WU	− 1.75 ± 1.68	− 6.4 ± 3.1	< 0.001
RVGLS*PAC, %*mmHg/ml	− 26 ± 16.7	− 75.1 ± 54.3	< 0.001
RVEF/PASP, %*mmHg	0.51 ± 0.21	1.02 ± 0.36	< 0.001
RVEF/PVR, %*WU	5.37 ± 4.16	15.72 ± 8.26	< 0.001
RVEF*PAC, %/mmHg/ml	85.3 ± 50.4	181.9 ± 133.1	0.006
TAPSE/RVSP (echo/echo)	0.30 ± 0.17	0.44 ± 0.21	0.012
TAPSE/RVSP (CMR/echo)	0.28 ± 0.16	0.55 ± 0.21	0.03
RV GLS/RVSP (CMR/echo)	− 0.22 ± 0.19	− 0.57 ± 0.21	0.03
RVEF/RVSP (CMR/echo)	0.68 ± 0.39	1.26 ± 0.58	0.01

*6MWT* six minute walk test distance, *BNP* serum brain natriuretic peptide, *BSA* body surface area, *CEP* composite end-point, *CI* cardiac index, *CMR* cardiac magnetic resonance, *echo* echocardiography, *GLS* global longitudinal strain, *FAC* fractional area change, *LV* left ventricle, *mPAP* mean pulmonary artery pressure, *PAC* pulmonary arterial compliance, *PASP* pulmonary artery systolic pressure, *PET* positron emission tomography, *PVR* pulmonary vascular resistance, *RV* right ventricle, *RVEF* right ventricle ejection fraction, *RVSP* right ventricle systolic pressure obtained by echocardiography, *SUV* standardized uptake value, *TAPSE* tricuspid annular plane systolic excursion, *WHO* World Health Organisation

**Fig. 3:**
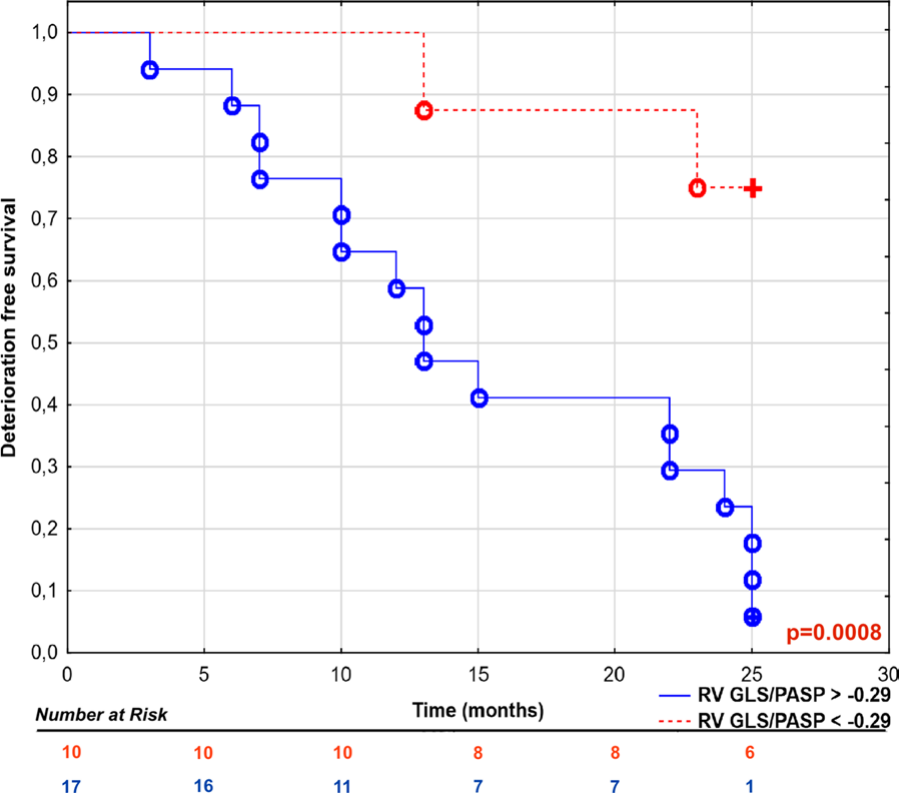
Kaplan–Meier curves presenting deterioration free survival in pulmonary artery hypertension (PAH) patients basing on RV GLS/PASP cut-off value, log-rank test, p = 0.0008. °—complete events, +—censored events

ROC analysis revealed highest area under the curve (AUC) in predicting CEP for RV GLS/PASP ratio, Table 4. Importantly, AUC for coupled parameter RV GLS/PASP was numerically higher than for RV GLS alone (0.96 [0.88–1] vs 0.87 [0.73–1], p = ns). Patients with RV GLS/PASP cut-off value (ROC analysis) higher than (−)0.29%/mmHg had worse prognosis, log-rank test, p = 0.0008, Fig. 3.

**Table 4:** Comparison of area under curve (AUC) of various parameters for prediction of composite end-point (ROC analysis)

Value	AUC (95% confidence interval)	p-value
RV GLS/PASP	0.96 (0.88–1)	p < 0.001
RV GLS/PVR	0.94 (0.86–1)	p < 0.001
RV GLS*PAC	0.89 (0.76–1)	p < 0.001
RV GLS	0.87 (0.73–1)	p < 0.001
SUV_RV/LV_	0.84 (0.67–1)	p < 0.001
RVEF	0.83 (0.65–1)	p < 0.001
PVR	0.88 (0.75–1)	p = 0.04
PAC	0.72 (0.52–0.92)	p = 0.03
TAPSE/RVSP (echo/echo)	0.79 (0.62–0.96)	p = 0.005
TAPSE/RVSP (CMR/echo)	0.76 (0.58–0.94)	p = 0.004
RV GLS/RVSP (CMR/echo)	0.89 (0.77–1)	p < 0.001
RVEF/RVSP (CMR/echo)	0.78 (0.58–0.96)	p = 0.002

*CMR* cardiac magnetic resonance, *echo* echocardiography, *GLS* global longitudinal strain, *LV* left ventricle, *PAC* pulmonary arterial compliance, *PASP* pulmonary artery systolic pressure, *PET* positron emission tomography, *PVR* pulmonary vascular resistance, *RV* right ventricle, *RVEF* right ventricle ejection fraction, *RVSP* right ventricle systolic pressure obtained by echocardiography, *SUV* standardized uptake value, *TAPSE* tricuspid annular plane systolic excursion

Furthermore, ROC analysis showed that also RV GLS*PAC has a comparable predictive value for the composite endpoint as RV GLS/PVR (p = 0.33) and numerically higher than PAC alone (0.89 vs 0.72, p = 0.18). Patients with an RV GLS*PAC value higher than (−)48.35%*mmHg/ml had significantly worse prognosis in Kaplan–Meier analysis, log-rank test, p = 0.004**.**

In univariable logistic regression analysis all three coupling parameters were significantly associated with CEP. However, multivariable stepwise regression analysis demonstrated that in the model predicting time to clinical end-point RV GLS/PASP remained statistically significant (Table 5). The model containing RV GLS/PASP beside other PAH prognostic parameters (WHO class, RVEF, mPAP, SUV_RV/LV_) had higher adjusted R^2^ than model with sole RV GLS (R^2^ = 0.68 vs 0.62).

**Table 5:** Univariable and multivariable logistic regression analysis for the composite endpoint

Value	Univariable analysis		
	HR	95% CI	p-value
WHO Class	12.34	1.53–95.49	0.01
Age	0.98	0.93–1.03	0.50
BSA	0.03	0.01–2.88	0.13
BNP	1.03	0.99–1.08	0.13
6MWT	0.99	0.97–1	0.05
*PET parameters*			
SUV_RV_/SUV_LV_ ratio	17.84	1.48–214.8	0.02
*CMR parameters*			
RVEF	0.83	0.71–0.96	0.01
RV mass/BSA	1.09	1.06–1.18	0.05
PAC	0.50	0.23–1.21	0.09
*RHC parameters*			
mPAP	1.67	1.04–1.30	0.008
PCWP	1.04	0.74–1.47	0.79
DPG	1.31	0.99–1.44	0.05
RAP	1.61	1.03–2.51	0.03
CI	0.24	0.05–1.07	0.06
PVR	1.56	1.08–2.25	0.01
SvO_2_	0.99	0.87–1.21	0.89
RV GLS	1.38	1.05–1.81	0.01
RV GLS/PASP*	19.88	3.59–1099	0.003
RV GLS/PVR	2.31	1.20–4.45	0.01
RV GLS*PAC	1.09	1.01–1.18	0.01

*6MWD* 6-minute walking test distance, *BSA* body surface area, *BNP* brain natriuretic peptide, *CI* cardiac index, *DPG* diastolic pulmonary gradient, *GLS* global longitudinal strain, *mPAP* mean pulmonary artery pressure, *PAC* pulmonary arterial compliance, *PASP* pulmonary artery systolic pressure, *PCWP* pulmonary capillary wedge pressure, *PET* positron emission tomography, *PVR* pulmonary vascular resistance, *RAP* right atrial pressure, *RV* right ventricle, *RVEF* right ventricle ejection fraction, *SUV* standardized uptake value, *SvO*_*2*_ mixed venous oxygen saturation, *WHO* World Health Organisation

*Remained statistically significant in multivariable stepwise regression model included PAH prognostic parameters (WHO class, SUV_RV_/SUV_LV_, RVEF, mPAP), (R^2^ = 0.68, p < 0.001)

We also performed similar sub analysis for RVEF as a surrogate of RV contractility. RVEF was significantly lower in CEP patients(40.9 ± 8.8 vs 52.5 ± 8.4, p = 0.004) and there were statistically significant differences in coupling parameters—RVEF/PASP; RVEF/PVR and RVEF*PAC between both groups, Table 3. Also, RVEF was a significant parameter in univariable regression and ROC analysis.

### Non-invasive coupling approach

Additional analysis of fully non-invasive coupling approach was also conducted, where TAPSE (derived from echo or CMR) was used as a surrogate of RV contractility and echo RV systolic pressure as a surrogate of RV afterload. There were significant differences in TAPSE/RVSP values between CEP(+) and CEP(−) groups. Similar results we also got with other non-invasive coupling parameters—RV GLS/RVSP (CMR/echo); TAPSE/RVSP (CMR/echo) and RVEF/RVSP (CMR/echo), Table 3. However, all non-invasive coupling parameters had lower AUCs in predicting CEP than RV GLS/PASP, Table 4.

## Discussion

This is the first study to our knowledge which: (1) confirms prognostic value of indexed CMR-derived RV GLS by RHC-derived variables describing afterload in PAH patients and (2) combines RV-arterial coupling parameters with metabolic alterations of RV myocytes using multimodal approach.

PAH is a condition associated with high mortality, even in young patients. The prognosis is strictly related to RV function, one of the main predictors of long-term outcome in patients with pulmonary hypertension irrespective of its etiology [20]. Our study population consisted of stable patients with mostly idiopathic, moderate-to-severe PAH (basing on PAP). Mean standard, hemodynamic, prognostic parameters (according to ESC guidelines) e.g. mPAP or cardiac index were overall unfavorable; whereas mean 6MWT distance or BNP levels were mostly in normal ranges or barely deteriorated. These suggest need for more accurate assessment and better prognostication of PAH patients with already moderate risk of death.

In PAH, RV load could increase even fivefold, thus coupling can only be achieved by an almost similar increase in contractility [6]. We hypothesized that quantification of RV-arterial coupling based on RV GLS as a contractility parameter may offer additive value. It was confirmed that RV strain mirrors RV-arterial uncoupling and its assessment provides information on the adaptation of RV inotropic function to afterload [13].

To obtain RV GLS, CMR feature tracking analysis was used. This method has been validated against myocardial tagging for LV strain analysis and recently against RV speckle tracking imaging for RV longitudinal strain evaluation in tetralogy of Fallot patients or arrhythmogenic RV dysplasia [14]. Systematic evaluation of RV strain in PH with CMR feature tracking seems to be feasible, reproducible and it was proven that reduced RV strain is associated with subsequent clinical deterioration. Our findings are also consistent with those reported before with the use of echocardiography—patients with worse prognosis had less negative RV GLS [21]. Haeck et al. evaluated 150 patients with PH and demonstrated that echo-derived RV GLS is a significant determinant of all-cause mortality risk compared to patients with higher GLS (threshold of <  − 19%) [12]. In our study the cut-off value was < − 16%. We are aware, that RV GLS represents only longitudinal shortening and when PAH advances, RV becomes also more hypertrophic and circumferential deformation becomes also important [12, 13]. However, RV GLS in our study strongly correlated with other RV systolic function parameters e.g. TAPSE or FAC. Research by di Siqueira et all proved that CMR derived RV GLS was independently associated with the CEP after adjustment for a few clinically meaningful covariates e.g. cardiac index [14].

In this study, we propose indexation of RV GLS with RHC-derived surrogates of RV afterload (PASP; PVR; PAC) as that may provide even more accurate estimation of RV-arterial coupling in PAH [4]. The RV is a pulsatile pump and its efficiency depends on sustained hemodynamic coupling with the compliant pulmonary circulation. The resistance and compliance of the pulmonary vasculature contribute to the RV afterload, which comprises the steady and unsteady loads that oppose the ejection of blood during ventricular systole. The RV afterload is lower than the LV afterload, both in terms of mean and pulsatile and its rapid changes e.g. during acute pulmonary embolism could be fatal for patient [15, 22]. There is a growing appreciation of the prognostic value of pulmonary arterial compliance in PAH and some evidence indicates PAC is a better predictor of outcomes than PVR. The most feasible option to obtain PAC is a ratio of stroke volume to pulmonary artery pulse pressure measured by RHC. Despite of the fact that this method overestimates real compliance, hence does not include amount of blood flow from the pulmonary circulation into the capillary bed during systole, it was proven as significant prognostic factor in PAH [23]. Interestingly, some studies confirmed that low PAC (and worse prognosis) was observed in PAH patients, even when PVR was normal. This suggest that PAC could be a cause rather than a consequence of distal small-vessel proliferative vasculopathy [12]. In our study, PAC did not correlate with neither 6MWT distance, BNP, nor RVEF. However, RV GLS*PAC ratio was in strong relationship with hemodynamic parameters, FDG uptake and prognosis. Our results showed that PAC only in combination with contractility parameter have prognostic value in stable PAH patients. This indicates the importance of the holistic approach for RV function assessment e.g. using coupling analysis.

In PAH decreased pulmonary artery compliance and increased pulse wave velocity create reflected waves which appear during mid or late systole, resulting in increased PASP, pulse pressure, and RV pulsatile afterload [3, 12]. The elevated PASP increases RV wall stress and oxygen consumption. This over time leads to RV hypertrophy and dilation, lower CO (due to RV–pulmonary artery uncoupling) and accompanying higher glucose demand of RV myocytes. In more severe cases areas of fibrosis or inflammation appear in RV insertion points that when assessed by late gadolinium enhancement indicate higher long term risk of adverse outcome [11]. In our study, the highest area under curve in ROC analysis for predicting clinical end-point had RV GLS/PASP. Although differences between this ratio and other proposed coupling parameters were not significant, only RV GLS/PASP was significantly associated with CEP in multivariable stepwise regression analysis.

RV-arterial coupling may also be presented with a use of other surrogates of RV contractility derived from CMR imaging, such as RVEF or SV divided by end-systolic volume – SV/ESV [24, 25]. Although, RVEF was statistically significant in univariable regression analysis for the CEP, none of RVEF-coupling parameters achieved statistically significant prognostic value. RVEF still remain one the most important prognostic factors in PAH [1], but it may be not the best surrogate in estimating RV-arterial coupling. Recent papers confirmed that increased pulmonary arterial pressures are associated with increases in FDG uptake and that patients with higher SUV_RV/LV_ had significantly worse prognosis [18, 26]. In our study, PAH patients had higher glucose uptake than control group and SUV_RV/LV_ significantly correlated with various hemodynamic parameters. Furthermore, in the current study we managed to confirm that worse values of coupling parameters are linked with altered glucose metabolism in PAH patients’ heart. This is in agreement with previous research, which suggested that if Ees/Ea ratio and its surrogates are out of normal ranges, decreased mechanical efficiency and loss of ventriculoarterial coupling is observed what requires the increased oxygen consumption [27]. This results in mitochondrial dysfunction and altered myocytes’ metabolism. Interestingly, among many parameters only RV GLS/PASP remained statistically associated with SUV_RV/LV_ dichotomized at 1 what suggests a strong relationship between declining RV-arterial coupling and altered RV myocytes’ metabolism. Both phenomena preceded clinical deterioration in hemodynamically stable PAH patients.

We are aware that glucose uptake measured using one tracer (FDG) in PET imaging does not fully reflects real “metabolic shift” of RV myocytes into glycolysis but SUV_RV/LV_ ratio parameter is now considered as an established surrogate in many papers [17, 18, 27]. Therefore, we would like to underline this possible connection between coupling parameters and glucose uptake. Subtle changes both in cardiac metabolism and RV-arterial coupling seem to be an interesting foundation for new research in this field.

## Limitations

The main limitations of this study are relatively small patient population and that only initial measurements at baseline visit were analysed. However, study group size is equivalent to similar previous surveys in this field. Coupling parameters, e.g. RV GLS/PASP, RV GLS/PVR and RV GLS*PAC ratios are simplified estimations and we did not use direct measurements from pressure–volume loops, but many previous studies used similar formulas and confirmed their significance in PAH patient evaluation [28]. Another limitation is that PET/CMR images were obtained in patients in stable condition, so the intrinsic diversity of the group is limited in comparison to the unselected patients seen in everyday practice.

## Conclusions

Multimodal approach (PET/CMR hybrid and heart catheterization) allows to confirm the relationship between RV-arterial uncoupling and higher metabolic demand that heralds the clinical deterioration of PAH patients. Coupling parameters like RV GLS/PASP may add additional prognostic value in PAH patients’ assessments but this requires further larger studies.

## Data Availability

The datasets generated and/or analyzed during the current study are available from the corresponding author on reasonable request.

## Abbreviations

6MWT: Six minute walk test
BNP: Brain natriuretic peptide
BSA: Body surface area
bSSFP: Balanced steady state free precession
CEP: Composite end-point
CMR: Cardiovascular magnetic resonance
CO: Cardiac output
dPAP: Diastolic pulmonary artery pressure
Ea: Arterial elastance
Ees: End-systolic elastance
ESC: European Society of Cardiology
FAC: Fractional area change
FDG: ^18^F-fluorodeoxyglucose
GLS: Global longitudinal strain
LV: Left ventricle/left ventricular
LVEF: Left ventricular ejection fraction
mPAP: Mean pulmonary artery pressure
PAC: Pulmonary artery compliance
PAH: Pulmonary arterial hypertension
PASP: Pulmonary artery systolic pressure
PAWP: Pulmonary artery wedge pressure
PET: Positron emission tomography
PH: Pulmonary hypertension
PVR: Pulmonary vascular resistance
RHC: Right heart catheterization
ROC: Receiver operator characteristic
RV: Right ventricle/right ventricular
RVEF: Right ventricle ejection fraction
sPAP: Systolic pulmonary artery pressure
SUV: Standardized uptake value
SV: Stroke volume
TAPSE: Tricuspid annular plane systolic excursion
WHO: World Health Organization
WU: Wood’s Units
